# Contact inhibition against senescence

**DOI:** 10.18632/oncotarget.2446

**Published:** 2014-09-05

**Authors:** Peiqing Sun

**Affiliations:** Departments of Cell and Molecular Biology, The Scripps Research Institute, 10550 N. Torrey Pines Rd., La Jolla, CA 92037

Cellular senescence is a form of irreversible growth arrest historically associated with the exhaustion of replicative potential of *in vitro* cultured cells. Senescence can be triggered by many stimuli, including telomere attrition, DNA damage, oxidative stress, activation of oncogenes, and inactivation of tumor suppressor genes, and plays important roles in tumor suppression, organism aging, tissue repair, and embryonic development [1,2].

One key feature of senescence is irreversibility. Once become arrested, senescent cells cannot be stimulated to proliferate by any known physiological stimuli, despite the availability of space, nutrients and growth factors. This is in contrast to quiescence resulted from growth factor deprivation, which is reversible upon replenishment of the missing growth factors. Another type of quiescence is contact inhibition, in which cells become growth arrested when they contact each other at high density. Similarly contact inhibition is also reversible, as cells re-enter cell cycle and start proliferating when split and re-plated at low density. It had been a mystery what signaling pathways differentiate irreversible senescence from reversible quiescence induced by growth factor deprivation or contact inhibition. Now a recent paper published by Blagosklonny's group demonstrates that the conversion of reversible cell cycle arrest to irreversible senescence, a process termed geroconversion, is governed by the AKT-mammalian target of rapamycin (mTOR) pathway [3].

The AKT/mTOR pathway is an important intracellular growth promoting pathway. Upon activation by receptor tyrosine kinases or G-protein coupled receptors, AKT activates mTOR, which in turn stimulates protein translation through inhibitory phosphorylation of eukaryotic translational initiation factor eIF4E-binding protein 1 (4E-BP1) that inhibits eIF4E, and activating phosphorylation of p70S6 kinase (S6K) that phosphorylates ribosomal S6 protein. A previous study suggests that the status of the mTOR differs between quiescence and senescence [4]. Whereas the phosphorylation of mTOR and its downstream substrates S6K and 4E-BP1 remains high in senescent cells, it is dramatically decreased in cells undergoing serum starvation-induced quiescence. Additional studies indicate that conditions that activate the mTOR pathway convert quiescence to senescence, and that those that inhibit mTOR (such as treatment with the mTOR inhibitor rapamycin and hypoxia) suppress the conversion from quiescence to senescence [4-7]. These studies suggest that the choice between senescence and quiescence is determined, at least in part, by the status of the mTOR pathway.

This new study published by the Blagosklonny group focuses on the difference between senescence and quiescence induced by contact inhibition [3]. The author first confirmed that contact inhibition was a reversible growth arrest by showing that the contact-inhibited cells restarted proliferation after splitting. The reversible contact inhibition was accompanied by suppression of the activating phosphorylation of AKT and the phosphorylation of ribosomal S6 protein in normal cell lines derived from multiple origins, including retinal pigment epithelial (RPE) cells, human WI38t fibroblasts, normal human bladder cells (NBCs) and rat intestinal epithelial IEC18 cells. These findings indicate that suppression of the AKT/mTOR pathway is a general phenomenon associated with contact inhibition in normal cells. To investigate whether the suppressed AKT/mTOR pathway contributes to the reversibility of contact inhibition, they designed shRNA that silenced the expression of TSC2, an AKT substrate that inhibits mTOR activity by suppressing the mTOR-activating small GTP binding protein Rheb. Silencing of TSC2, which leads to reactivation of mTOR, prevented the resumption of proliferation of contact-inhibited cells upon replating at low density, and rendered these cells positive for the senescence-associated β-galactosidase marker. Therefore, reactivation of the mTOR pathway converts reversible quiescence to irreversible senescence, suggesting that low mTOR pathway activity is required for maintaining a reversible quiescence program during contact inhibition.

The authors then performed a reciprocal experiment by asking whether contact inhibition-mediated suppression of mTOR could convert senescence into reversible quiescence. To this end, a cancer cell line HT-p21 was used which ectopically expresses a key senescence effector p21 in an isopropyl-thio-galactosidase (IPTG)-dependent fashion. Addition of IPTG induces p21, leading to senescence in these cells. As HT-p21 is a cancer cell line that do not undergo contact inhibition, the authors cocultured HT-p21 cells with confluent RPE cells, thus creating an artificial contact-inhibition condition, which effectively inhibited mTOR activity, as compared to the HT-p21 cells cultured alone. Importantly, HT-p21 cells induced to overexpress p21 by IPTG in the co-culture with confluent RPE cells did not display senescence morphology, and retained proliferative potential in that they resumed proliferation and formed colonies upon IPTG removal and replating at low density. In contrast, IPTG-treated HT-p21 cells cultured alone became senescent and failed to proliferate or form colonies after replating. Taken together, these findings demonstrate that suppression of the mTOR pathway during contact-inhibition is responsible for the maintenance of the reversible growth arrest state and prevents the induction of irreversible senescence. Supporting the key role of mTOR in differentiating the quiescence and senescence states, the authors showed that other conditions that inhibit mTOR, such as treatment with rapamycin and exhaustion of nutrients in the medium by a high-density culture, also suppress the induction of senescence.

The finding that contact-inhibited cells do not undergo senescence may have important physiological implications. Most cells in an organism are contact-inhibited and thus should retain the ability to resume proliferation under conditions such as tissue damage, where the tissue integrity needs to be restored. In addition, suppression of senescence in a densely packed tumor may explain why tumor cells are sometimes resistant to anti-cancer drugs. This study also raises several intriguing questions that need to be answered by further experiments. For example, what is the mechanism underlying mTOR suppression in contact inhibition? What act downstream of mTOR to promote quiescence-to-senescence conversion? Does the mTOR status differentiate senescence and quiescence under physiological or pathological conditions in vivo?
